# Report of a case combining solitary Peutz-Jeghers polyp, colitis cystica profunda, and high-grade dysplasia of the epithelium of the colon

**DOI:** 10.1186/s12957-017-1253-x

**Published:** 2017-10-18

**Authors:** Alexandros Papalampros, Michail G. Vailas, Maria Sotiropoulou, Efstratia Baili, Spiridon Davakis, Demetrios Moris, Evangelos Felekouras, Ioanna Deladetsima

**Affiliations:** 1 1st Surgical Department, Athens University School of Medicine, “Laiko” General Hospital, Agiou Thoma 17, 11527 Athens, Greece; 2 Pathology Department, Athens University School of Medicine, “Laiko” General Hospital, Agiou Thoma 17, 11527 Athens, Greece

**Keywords:** Colitis cystica profunda, Hamartomatous polyps, High-grade dysplasia, Peutz-Jeghers-type polyp, Solitary Peutz-Jeghers polyp

## Abstract

**Background:**

Colitis cystica profunda is a rare nonneoplastic disease defined by the presence of intramural cysts that contain mucus, usually situated in the rectosigmoid area, which can mimic various malignant lesions and polyps. Its etiology still remains not fully elucidated, and several mechanisms such as congenital, post-traumatic, and infectious have been implicated in the development of this rare entity.

**Case presentation:**

Herein, we describe a unique case of colitis cystica profunda in the setting of Peutz-Jeghers-type polyp of the sigmoid colon, associated with high-grade dysplasia of the overlying epithelium in a 48-year-old female patient, who presented to the emergency room with signs of intestinal obstruction. To the best of our insight, this is the first manifestation ever reported in the literature regarding the coexistence of solitary Peutz-Jeghers-type polyp, colitis cystica profunda, and high-grade dysplasia of the epithelium of the colon.

**Conclusions:**

The purpose of this case report is to highlight colitis cystica profunda and its clinical significance. An uncommon nonneoplastic entity, many times masquerading as malignant lesion of the rectosigmoid area of the colon. Clinicians and pathologists should be aware of this benign condition that is found incidentally postoperatively in patients undergoing colectomies, leading to unnecessary increase of morbidity and mortality in these patients, who otherwise could have been cured with conservative treatment only.

## Background

Colitis cystica profunda (CCP) is generally an uncommon benign condition of the colon and rectum that can resemble and impersonate different conditions and diseases such as mucinous adenocarcinomas, carcinoid tumors, and pancreatic heterotopias [1]. Less than 200 cases have ever been investigated in the medical literature [2]. Histologically, it is comprised of numerous intramural or submucosal mucous containing cysts. The most frequent sites of appearance are in the rectum, usually 6–7 cm from the anal verge and sigmoid colon [3]. Its etiology is not fully elucidated with many authors proposing ischemic, inflammatory, and post-traumatic mechanisms of development [4]. In 1957, Goodall and Sinclair published the first modern description in the English medical literature of this benign condition, a disease first described by Stark in 1766, who found these lesions in after death examinations of cases of dysentery [1, 5, 6]. The term “colitis cystica profunda” was presented later on in 1863 by Virchow, accompanying an article describing multiple polypoid cystic submucosal lesions [6, 7]. Knowing the existence of this pathological entity is of great importance since it can emulate clinically and histologically a malignant lesion of the colon, leading to unnecessary operative management [1, 6, 8]. Solitary Peutz-Jeghers-type polyp is an uncommon hamartomatous lesion without associated mucocutaneous pigmentation, any other gastrointestinal polyp or a family history of Peutz-Jeghers syndrome [9–11]. To the best of our insight, its association with CCP and dysplastic changes in the overlying epithelium has never been reported again in the English scientific writings. Herein, we demonstrate a case of a 48-year-old female patient who presented to the emergency department suffering from longstanding intermittent rectal bleeding, abdominal pain, and distension.

## Case presentation

**Fig. 1:**
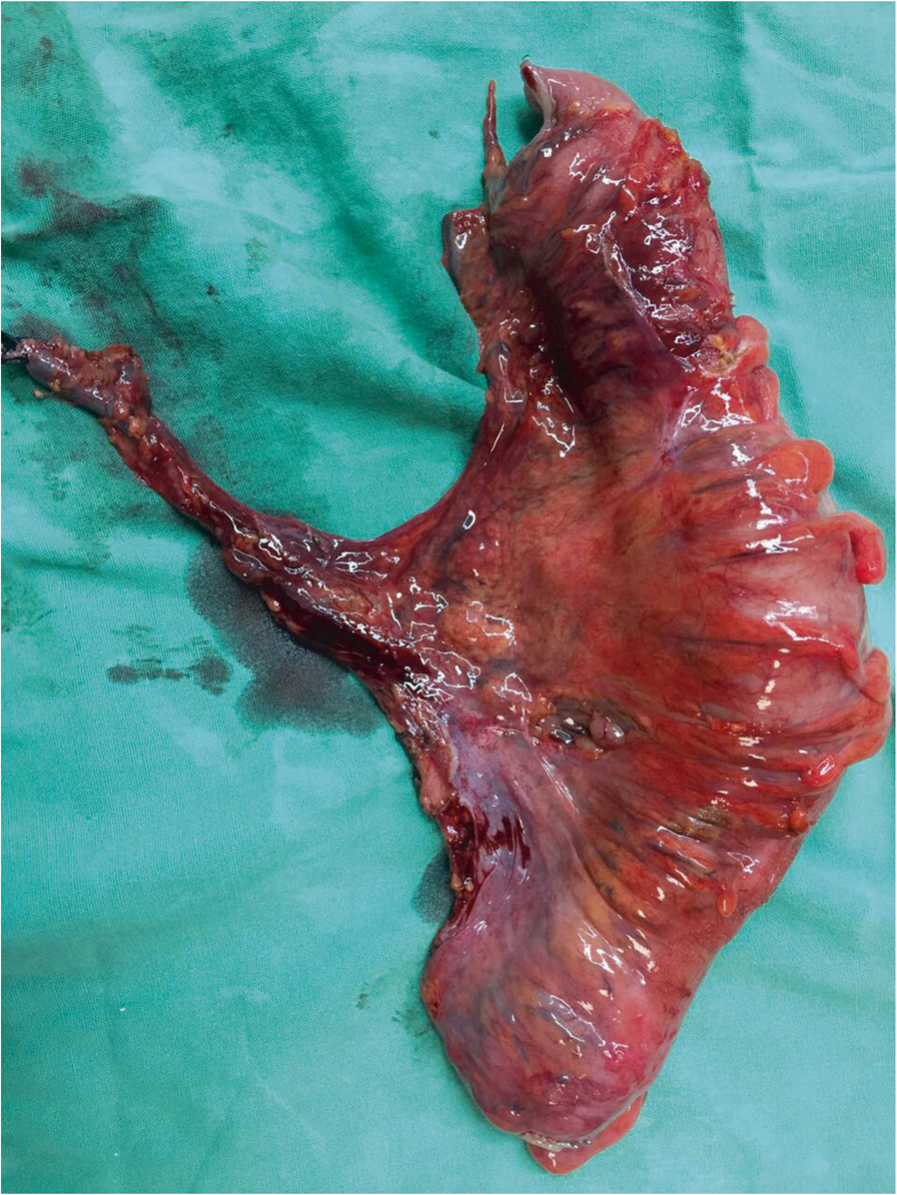
The resected bowel specimen

A previously healthy 48-year-old woman, with no significant past medical history and no family history of colorectal diseases, presented to the emergency room with abdominal distension, colicky pain, and a history of repeated episodes of lower gastrointestinal hemorrhage for as long as couple of months. She also complained of 8 kg weight loss in the last 2 months. She denied taking any prescription or over-the-counter medications. Clinical examination revealed a malnourished woman in severe distress due to diffuse abdominal tenderness. Signs of colonic obstruction were apparent. Digital rectal examination was indicative for rectal bleeding. Her vital signs were temperature 38°C, pulse 118/min, blood pressure 105/60 mmHg, and respiratory rate 20/min. Blood counts showed Hb 7.4 g/dl and a white blood count of 16.000. Serum electrolytes, liver function tests, and urinalysis were unremarkable. A plain abdominal X-ray and abdominal CT scan confirmed the signs of large bowel obstruction, along with a polypoid mass fully occluding the lumen of the terminal sigmoid colon. The patient was taken to the operating room where a standard left hemicolectomy was performed because of the malignant appearance of the mass and the extensive lymph node involvement that was found intraoperatively (Fig. 1).

**Fig. 2:**
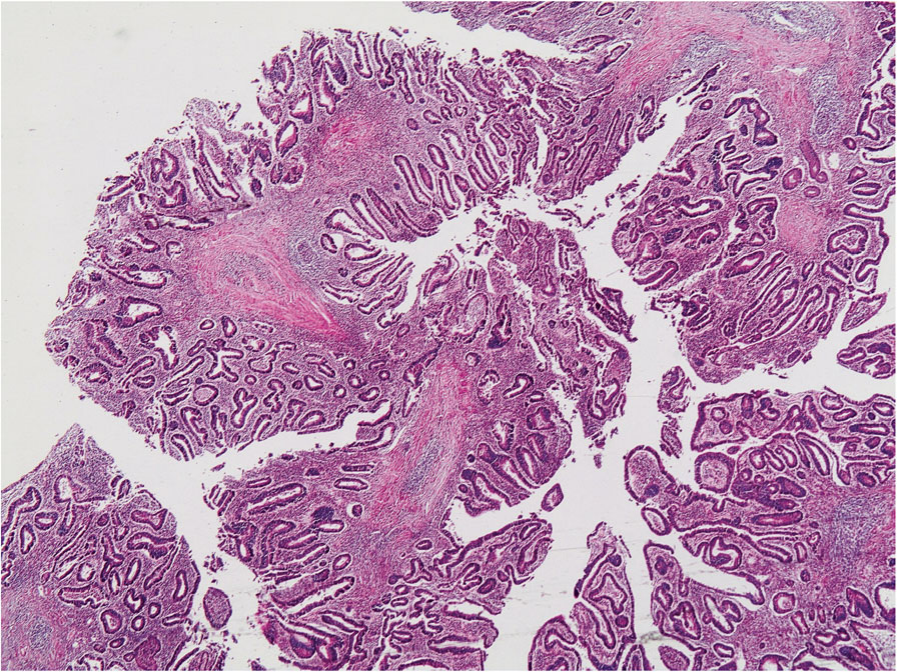
Microscopic examination, revealing mucosa projections with a central muscular core (hematoxylin and eosin × 20)

**Fig. 3:**
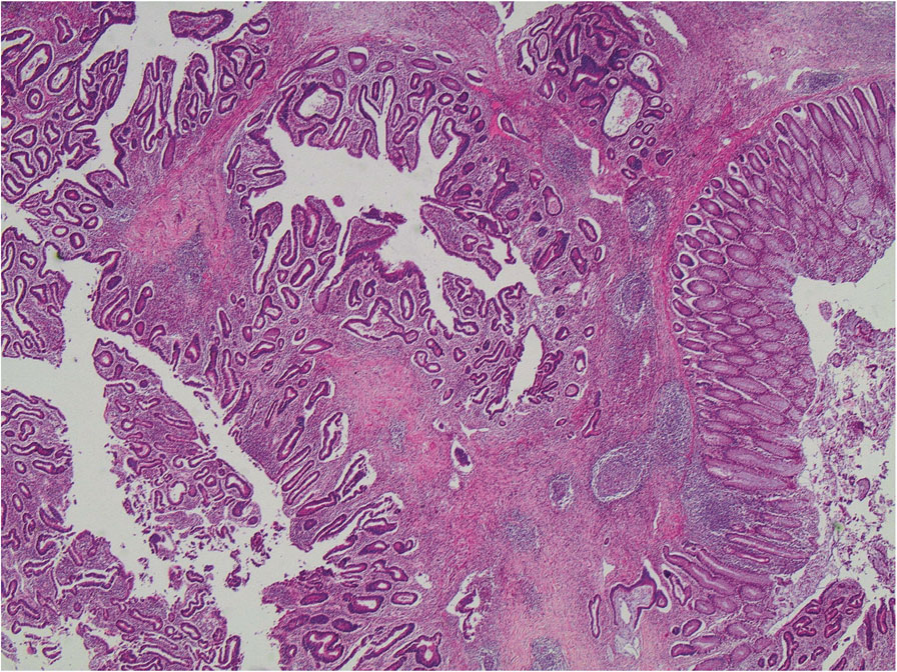
Biopsy of the lesion showing misplaced mucosa, forming cystic structures (hematoxylin and eosin × 40)

**Fig. 4:**
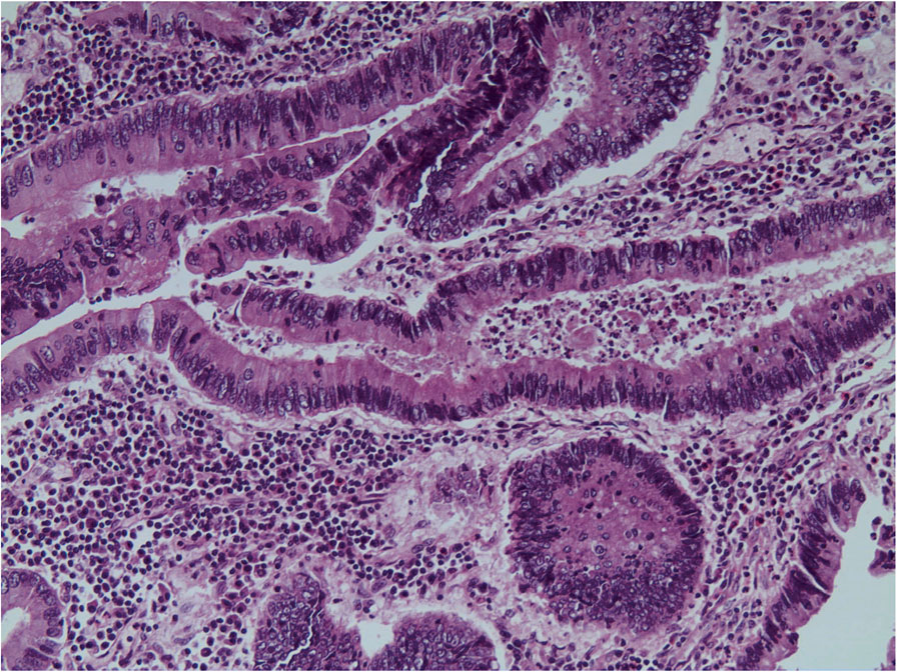
Biopsy of the lesion showing high-grade epithelial dysplasia of the overlying epithelium (hematoxylin and eosin × 400)

Histological examination revealed a branching polypoid lesion characterized by mucosa projections with a central muscular core (Fig. 2). Additionally, misplaced mucosa was encountered within the submucosa forming cystic structures, while partly preserved continuity with the polypoid part of the lesion could be demonstrated (Fig. 3). The colonic epithelium both of the exophytic and the endophytic component showed extensive adenomatous transformation with high-grade dysplasia (Fig. 4). A lesion-restricted transmural Crohn-like inflammation with prominent lymphoid aggregates was also present. The final diagnosis was consistent with a solitary hamartomatous polyp of Peutz-Jeghers type characterized by an inverted component, analogous to colitis cystica profunda, and by extensive high-grade dysplastic changes. The patient had an uneventful postoperative recovery and complete resolution of her symptoms. Follow-up colonoscopy 3 months after surgery showed no abnormal findings. The patient remains free of symptoms.

## Discussion

CCP is an uncommon entity, usually encountered in the third and fourth decades of life that has been gradually gaining recognition through sporadically reported cases in the literature. The initial description of this kind of lesions was made in 1766 by Stark, who discovered mucinous containing cysts in the colorectum of two patients; believed to have died of dysentery. Virchow subsequently brought in the term CCP in a case of submucosal cysts presenting as multiple polypoid lesions. Its clinical significance lies in the fact that it can often mimic cancerous and neoplastic masses of the colon and rectum, to which it must be recognized from [1–4].

In histological examination, CCP is a nonmalignant lesion defined by submucosal mucin-occupying cysts of variable size, which are covered by an epithelium with no cellular atypia and extend below the muscularis mucosa, intruding into the muscularis propria of the colonic wall in many cases. Furthermore, the surrounding connective tissue may show changes indicative of chronic inflammation and fibrosis. CCP may demonstrate itself in a focalized form or in a more disseminated pattern, with variable length of the wall of the bowel affected. The former type has been associated with diseases like solitary rectal ulcer syndrome, prolapse of the rectum, while the latter involves patients with inflammatory bowel disease, infectious, and radiation colitis [1, 5, 6].

The exact etiology and pathogenesis of this condition remains sharply defined; however, an unusual mucosal repair, with herniation of the gland epithelium into the submucosa in response to congenital, inflammatory, post-traumatic, or infectious processes has been suggested [4]. In any case, the rarity and uncommonness of this condition suggests that there may be unknown and obscure mechanisms contributing to the appearance and development of these lesions.

Notwithstanding etiology, it is of vital significance to differentiate CCP from invasive mucus-secreting adenocarcinoma. Keeping that in mind, a careful appraisal of both cytological and architectural features is imperative in pathological specimen [4, 6]. Commonly, the lack of cytological atypia and desmoplastic stromal reaction, along with the presence of lamina propria in histological analysis, are components that support the finding against a neoplastic procedure in cases of CCP. On the contrary, the existence of glandular epithelium with dysplasia deep to the muscularis mucosae in the bowel wall usually suggests the diagnosis of invasive adenocarcinoma, until proven otherwise. Therefore, pathologists require high level of suspicion to settle on the correct choice, analyze effectively, make the right decision, and diagnose correctly the disease since there may be dysplastic or neoplastic changes in the overlying epithelium of CCP patients, making the diagnosis troublesome and challenging [1, 6, 12].

CCP has been associated with adenocarcinoma of the colon in a number of cases [3]. Burt [13] described a patient with concurrent CCP and adenocarcinoma in the same mass of the sigmoid, accompanied 12 months later by operation for resection of the transverse colon for a lesion that had features of CCP only. Bhuta [14] also reported a case of CCP with coexisting carcinoma in situ. Mitsunaga et al. [15] recently published an article reporting a single polypoid CCP mass in relationship with malignant features.

Frequent and well known symptoms of CCP incorporate hematochezia, mucus secretion in feces, rectal tenesmus, altered bowel habits, and obstructive defecation. Intestinal obstruction such as in our case is rare [1, 4, 5]. Barium studies may show normal or nonspecific signs and features in the early stages or reveal narrowing of the lumen. Endoscopy findings are polypoid lesions covered by normal, edematous, or ulcerated mucosa. Neither barium enema nor colonoscopy may not be useful to distinguish benign from malignant masses in this setting. Endoscopic ultrasound can demonstrate a hypoechoic aggregate usually in the submucosa with no encompassing further infiltration or local lymph node involvement. Computed tomography and magnetic resonance imaging can aid to the diagnosis of CCP by revealing and showing submucosal cystic lesions with loss of perirectal fatty tissue and thickening of the levator ani muscular tissues [1, 3, 4]. In MRI, the submucosal cysts appear hyperintense on T2-weighted images. Anorectal physiology studies may help to elucidate the underlying pathophysiology such as solitary rectal ulcer. However, only in 54% of localized CCP cases were found to suffer from rectal prolapse [1, 8].

Currently, the treatment of CCP follows generally a conservative approach, and surgery is only justified when obstructive symptoms due to large lesions occur, in cases of chronic gastrointestinal hemorrhage or in protracted cases of rectal prolapse [1–6]. First-line management option incorporates dietary and lifestyle alterations to correct constipation and avoid straining. During the course of the disease, bulking laxatives, stool softeners, lubricants, and glucocorticoid enemas may be used. Bowel biofeedback therapy may be utilized with success in many individuals [1, 8].

Peutz-Jeghers Syndrome (PJS) is an infrequent syndrome with autosomal dominant inheritance, linked to a germline mutation of serine threonine kinase 11, which was initially identified by Peutz in 1921. It is described by gastrointestinal hamartomatous polyps, usually encountered in the small intestine along with mucocutaneous pigmentation. A Peutz-Jeghers-type polyp in a patient that is not accompanied with mucocutaneous pigmentation and family history of Peutz-Jeghers syndrome is termed solitary Peutz-Jeghers-type polyp [9, 10]. According to the medical literature, solitary hamartomatous polyp may be a variant or a separate disease entity. In comparison with PJS, Peutz-Jeghers-type polyps are associated with a lower risk of malignancy. This condition is extremely rare with a reported incidence of around 1:120,000. The most common location of these lesions is the small bowel [9–12]. Pathological examination reveals a characteristic tree-like branching of smooth muscle fibers, with a central core of smooth muscle covered by mucosa of near normal appearance. These polyps are best treated via methods of endoscopy or surgical operation depending on the size, the depth of the lesion, and associated malignant transformation on initial endoscopic biopsy [9, 10]. Its association with CCP has never been reported before in the medical literature.

In summary, a case of solitary Peutz-Jeghers-type polyp associated with CCP and high-grade dysplastic changes is reported herein. To our knowledge, this is the first case ever reported. The purpose of this case report is to highlight CCP and its clinical significance; an uncommon nonneoplastic entity, many times masquerading as malignant lesion of the rectosigmoid area of the colon. Clinicians and pathologists should be aware of this benign condition that is found incidentally postoperatively in patients undergoing colectomies, leading to unnecessary increase of morbidity and mortality in these patients, who otherwise could have been cured with conservative treatment only.

## Conclusions

Knowledge of CCP and its clinical presentation are indispensable to differentiate this benign entity from malignancy. However, there are cases in which this uncommon entity overlaps with dysplastic changes of the epithelium of the bowel, a fact that needs extra attention not only from the medical practitioners but also from the pathologists who examine the histology of the disease. Moreover, the association with other rare entities like solitary Peutz-Jeghers-type polyp, as in our case, is of paramount importance in order to avoid overly aggressive treatment decisions. Based on the current medical literature, our reported case is unique, highlighting the potential association of CCP with dysplasia along with the coexistence of solitary hamartomatous polyp; an association that has never been reported in the past.

## Abbreviations

CCP: Colitis cystica profunda
PJS: Peutz-Jeghers syndrome

## References

[1] Ayantunde AA, Strauss C, Sivakkolunthu M, Malhotra A (2016). Colitis cystica profunda of the rectum: an unexpected operative finding. World J Clin Cases.

[2] Dolar E, Kiyici M, Yilmazlar T, Gürel S, Nak SG, Gülten M (2007). Colitis cystica profunda. Turk J Gastroenterol.

[3] Spicakova K, Pueyo BA, de la Piscina PR, Urtasun L, Ganchegui I, Campos A, Estrada S, García-Campos F. Colitis cystica profunda: a report of 2 cases with a 15- year follow-up. Gastroenterol Hepatol. 2016;40:406-08. 10.1016/j.gastrohep.2016.04.011 27260634

[4] Madan A, Minocha A (2002). First reported case of colitis cystica profunda in association with Crohn’s disease. Am J Gastroenterol.

[5] Lord A, Hompes R, Arnold S, Venkatasubramaniam A (2015). Ultra-low anterior resection with coloanal anastomosis for recurrent rectal prolapse in a young woman with colitis cystica profunda. Ann R Coll Surg Engl.

[6] Hernandez-Prera JC, Polydorides AD (2014). Colitis cystica profunda indefinite for dysplasia in Crohn disease: a potential diagnostic pitfall. Pathol Res Pract.

[7] Guest CB, Reznick RK (1989). Colitis cystica profunda. Review of the literature. Dis Colon Rectum.

[8] Inan N, Arslan AS, Akansel G, Anik Y, Gürbüz Y, Tugay M (2007). Colitis cystica profunda: MRI appearance. Abdom Imaging.

[9] Limaiem F, Bouraoui S, Lahmar A, Jedidi S, Aloui S, Korbi S, Mzabi S (2011). Adenomatous transformation in a giant solitary Peutz-Jeghers-type hamartomatous polyp. Pathologica.

[10] Rathi CD, Solanke DB, Kabra NL, Ingle MA, Sawant PD (2016). A rare case of solitary Peutz Jeghers type hamartomatous duodenal polyp with dysplasia!. J Clin Diagn Res.

[11] Lunca S, Porumb V, Velenciuc N, Ferariu D, Dimofte G (2014). Giant solitary gastric Peutz-Jeghers polyp mimicking a malignant gastric tumor: the largest described in literature. J Gastrointestin Liver Dis.

[12] Silver H, Stolar J (1969). Distinguishing features of well differentiated mucinous adenocarcinoma of the rectum and colitis cystica profunda. Am J Clin Pathol.

[13] Burt CA, Handler BJ, Haddad JR (1970). Colitis cystica profunda concurrent with and differentiated from mucinous adenocarcinoma: report of a case. Dis Colon Rectum.

[14] Bhuta I, Prathikanti V (1976). Colitis cystica profunda. South Med J.

[15] Mitsunaga M, Izumi M, Uchiyama T, Sawabe A, Tanida E, Hosono K, Abe T, Shirahama K, Kanesaki A, Abe M (2009). Colonic adenocarcinoma associated with colitis cystica profunda. Gastrointest Endosc.

